# Does Preoperative Halo-Gravity Traction Reduce the Degree of Deformity and Improve Pulmonary Function in Severe Scoliosis Patients With Pulmonary Insufficiency? A Systematic Review and Meta-Analysis

**DOI:** 10.3389/fmed.2021.767238

**Published:** 2021-11-25

**Authors:** Zhao Yang, Yang Liu, Longtao Qi, Shanshan Wu, Jingwen Li, Yu Wang, Bin Jiang

**Affiliations:** ^1^Peking University First Hospital, Beijing, China; ^2^School of Pharmaceutical Sciences, Peking University Health Science Center, Beijing, China; ^3^Department of Orthopedics, Peking University First Hospital, Beijing, China; ^4^Department of Clinical Epidemiology and EBM, National Clinical Research Center for Digestive Diseases, Beijing Friendship Hospital, Capital Medical University, Beijing, China

**Keywords:** scoliosis, halo-gravity traction, pulmonary function, pre-operative, meta-analysis

## Abstract

**Background:** Halo-gravity traction is a commonly used clinical intervention to reduce surgical risk in patients with scoliosis before surgical correction. Some previous studies have focused on the application of halo-gravity traction on patients with severe spinal deformity and pulmonary insufficiency, but the overall effect of halo-gravity traction has not been fully understood. The object of the present study was to perform a meta-analysis exploring the efficacy of preoperative halo-gravity traction on radiographic measurement and pulmonary function in severe scoliosis patients with pulmonary insufficiency.

**Methods:** We searched the medical works of literature completed before January 17, 2021, in the databases of Pubmed, Embase, and Cochrane Library. Studies that quantitatively analyzed the effects of halo-gravity traction on the deformity and pulmonary functions of patients with severe scoliosis were included. Two researchers independently conducted the literature search, data extraction, and quality assessment. We used the Review Manager Software (version 5.4) for statistical analysis and data analysis. Mean difference (MD) with 95% confidence intervals (CIs) were calculated to evaluate the effects of halo-gravity traction.

**Results:** Seven studies involving 189 patients received halo-gravity traction therapy preoperatively were analyzed in our study. Preoperative halo-gravity traction significantly ameliorated the degree of deformity in severe scoliosis patients with pulmonary insufficiency, especially reduced coronal Cobb angle and sagittal Cobb angle effectively [mean deviation (MD) = 2 7.28 (95%CI 21.16–33.4), *p* < 0.001; MD = 22.02 (95%CI 16.8–27.23), *p* < 0.001]. Preoperative halo-gravity traction also improved the pulmonary functions in patients, especially increasing %FVC and %FEV1 [MD = −0.0662 (95%CI −0.0672–−0.0652), *p* < 0.001; MD = −0.0824 (95%CI −0.0832–−0.081)*, p* < 0.001].

**Conclusions:** Preoperative halo-gravity traction for severe scoliosis patients shows significant improvement in the degree of deformity and pulmonary functions. Halo-gravity traction is an effective method to improve the tolerance of patients to surgery in the perioperative period.

## Background

Spinal deformity directly affects the appearance of patients, their cardiopulmonary function, and their quality of life, especially in patients with severe scoliosis complicated with cardiopulmonary dysfunctions. For those patients, long operation time, large intraoperative and postoperative blood loss makes it difficult to perform surgical correction on them (1). Direct one-stage treatment of orthopedic surgery greatly increases the incidence of complications, mortality, and neurologic risk (2, 3), current clinical experience suggests that preoperative halo-gravity traction combined with staged surgery has become a widely accepted treatment with many advantages (4, 5).

Halo-gravity traction, also known as halo-wheelchair traction, is a widely used traction method in clinical practice which uses the own weight of patients as a reaction to achieve sustained traction. Halo-gravity traction not only gradually improves the coronal and sagittal deformity of patients but also extends their spine and improves pulmonary functions. The biggest advantage of halo-gravity traction is that its devices do not contain any femoral nails or pelvic nails, which reduces screw track-related complications. Moreover, patients treated with halo-gravity traction do not need long-term bed rests, and the incidence of bed-related complications such as bedsore and respiratory tract infection is significantly reduced (4, 5).

Previous studies have focused on the effects of preoperative halo-gravity traction on improving lung functions and the degree of deformity in severe scoliosis patients, but most of them failed to reach a clear conclusion. Thus, we conducted this study to analyze the effect of preoperative halo-gravity traction on the improvements of the degree of deformity as well as pulmonary functions in severe scoliosis patients with pulmonary insufficiency.

## Methods

### Search Strategy

Two independent researchers searched for published studies in the electronic databases of PubMed, Embase, and Cochrane Library. We searched all the studies from inception to January 17th, 2021 with no language restrictions. Our search strategies included Medical Subject Headings (MESH) – “scoliosis” and text. The three main searches were scoliosis, lung function, and halo-gravity traction. Detailed search strategies were listed in Appendix A–C.

### Study Selection

Two investigators independently screened all studies using the following criteria: studies that met both of the following criteria were included: ① case series; ②sample size was clear and the radiographic and pulmonary data of patients were available. Studies that met any of the following criteria were excluded: ① non-Chinese or English articles; ② full-text article could not be obtained; ③ pulmonary function data were not referred to in the article. Disagreements in study selection were solved by re-reading and discussion.

### Data Extraction

All data were extracted independently by two researchers and cross-checked. The extracted data mainly included: ① study characteristics: first author, publication year, study design, number of patients, etc.; ② baseline characteristics of the study subjects, including age, gender, halo-gravity traction period and maximum traction weight; ③ the outcome of interest: coronal Cobb angle, sagittal Cobb angle, FVC predicted value, FEV1 predicted value, coronal balance, sagittal balance, T1-S1 length, PEF, FVC, FEV1, FVC/FEV1, and FEV1/FVC.

### Quality Assessment and Risk of Bias

We referred to a checklist that was recommended by the Agency for Healthcare Research and Quality (AHRQ) and developed four criteria to assess the quality of the included studies (6). The four criteria were: ① defining the source of information; ② listing inclusion and exclusion criteria; ③ indicating time period used for identifying patients; ④ explaining any patient exclusions from the analysis. Each criterion was described as “Yes” or “No” or “Unknown” according to each study.

### Statistical Analysis

We used the Review Manager Software (version 5.4) for meta-analysis. Mean difference (MD) was used to assess the continuous outcomes of the effect of halo-gravity traction on the deformity of patients and pulmonary functions with a 95% CI. The generic inverse variance method was used to calculate the weight of each included study. The heterogeneity between studies was analyzed by chi-square test with Cochrane Q statistic. Besides, *I*^2^ statistic was used to measure heterogeneity quantitatively. An *I*^2^ ≥ 50% was considered a significant degree of heterogeneity and a random-effects model was applied to conduct the meta-analysis, whereas an *I*^2^ < 50% was considered irrelevant heterogeneity, and a fixed-effects model was applied. Values of *P* < 0.05 were considered statistically significant.

## Results

### Study Characteristics

**Figure 1:**
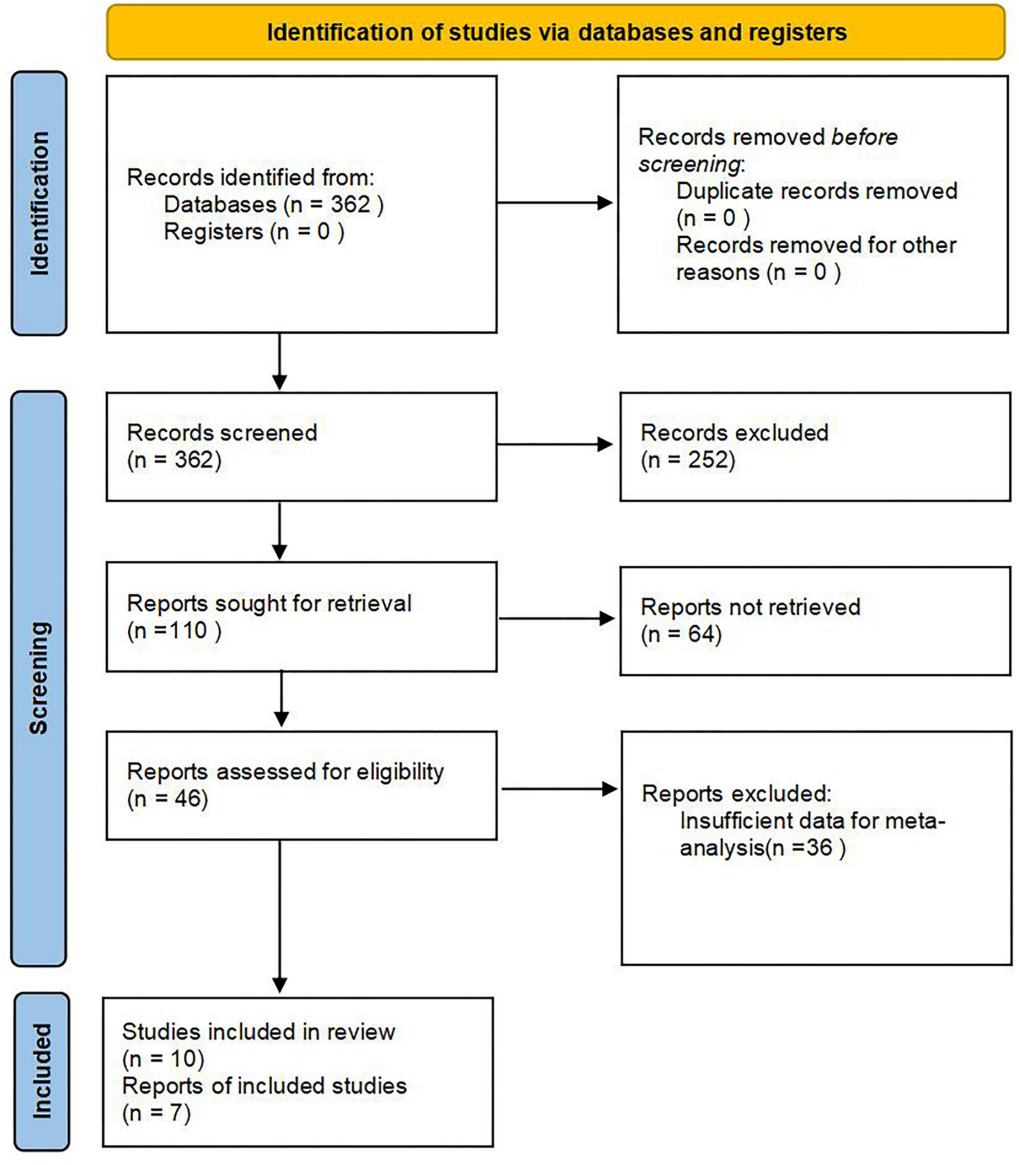
Flow diagram of the study selection process.

After thorough search and screening, a total of 10 articles providing radiographic and pulmonary function data pre- and post- halo-gravity traction were included; among which, seven studies were preserved for meta-analysis (7–13). The article selecting process is shown in Figure 1. The details and patient demographics for the seven included studies were summarized in Table 1.

**Table 1:** Summary of the included studies.

**References**	**Study design**	***n***	**Male patients**	**Female patients**	**Age (Years)**	**Traction period**	**Maximum traction weight**
Shimizu et al. (13)	Retrospective	18	11	7	33.0 ± 17.9	(28.2 ± 14.4) days	Ranged from 25 to 40 lb (20.4–50.1% of body weight)
Shi et al. (12)	Retrospective	35	21	14	14.9 ± 4.8	(72.3 ± 11.2) days	Initially 3–4 kg, which was added by 2 kg per day. The target weight was 30–50% of the body weight depending on patients' tolerance and should be no more than 15 kg
Iyer et al. (8)	Retrospective	30^a^	15	15	9.0 ± 2.3	(79 ± 43) days	Traction was started at 20% body weight and was increased to 50% of body weight by 4 weeks (increased at ~10% per week as tolerated)
Liu et al. (11)	Retrospective	29^b^	17	12	13.7 ± 2.9	(10.2 ± 6.6) weeks	(12.2 ± 2.8)kg
Li et al. (10)	Retrospective	11	4	7	18.82 ± 7.65	(9.55 ± 1.57) weeks	(49.95 ± 5.17) % of body weight
Koller et al. (9)	Retrospective	45	14	31	24 ± 14	(30 ± 14) days	(33 ± 9) % of body weight
Bao et al. (7)	Retrospective	21	7	14	26.2	76.2 days	13.6kg

a *Pre- and post-traction pulmonary function data were obtained in 23 patients*.

b *Because four patients were too young to be tested for pulmonary functions, only 25 of the patients were tested for pulmonary functions*.

### Quality Assessment

As a result of the quality assessment, all the studies defined the source of information; three studies did not list clear inclusion and exclusion criteria of patients; all studies except two indicated time periods used for identifying patients; three studies did not explain patient exclusions from the analysis. The included studies in this systematic review could be included to have high quality, overall. The results of the quality assessment were shown in Appendix D (Table A1).

### Radiographic Measurement

**Figure 2:**
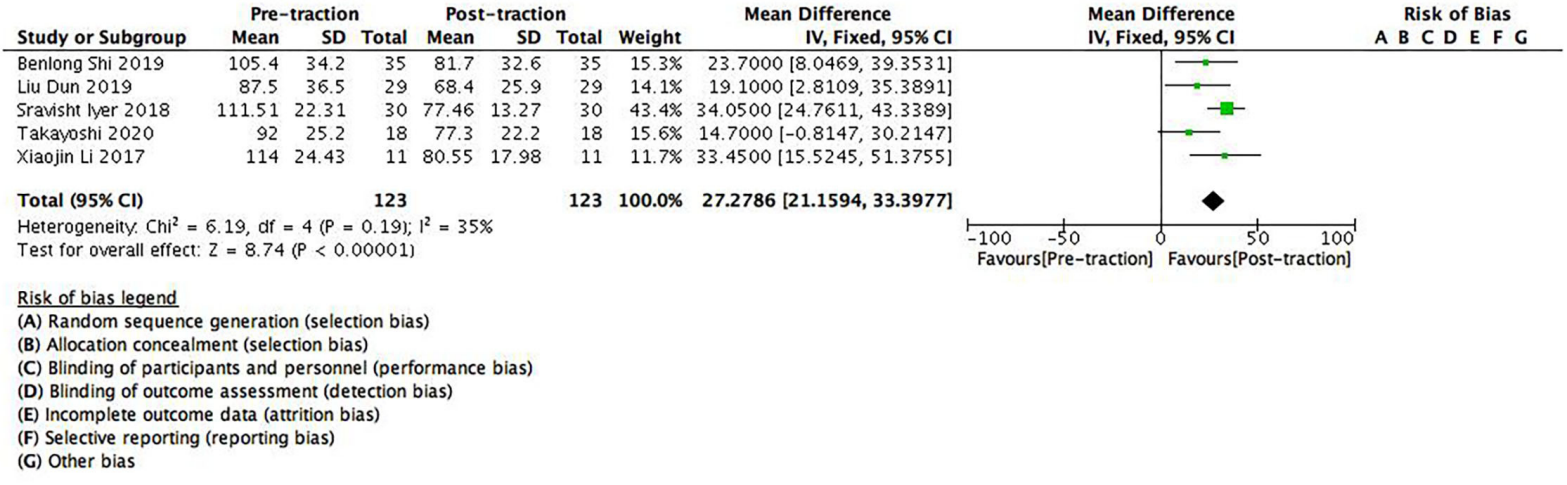
A forest plot depicting the changes in coronal Cobb angle of scoliosis patients between pre- and post-traction measurements.

The data of pre- and post-traction coronal Cobb angle was recorded in five studies containing 123 patients. Significant differences were found between pre- and post-traction coronal Cobb angle value [MD = 27.28 (95%CI 21.16–33.4), *p* < 0.001, *I*^2^ = 35%, Figure 2]. As minor heterogeneity was found between the studies, a fixed-effects model was used for this parameter.

**Figure 3:**
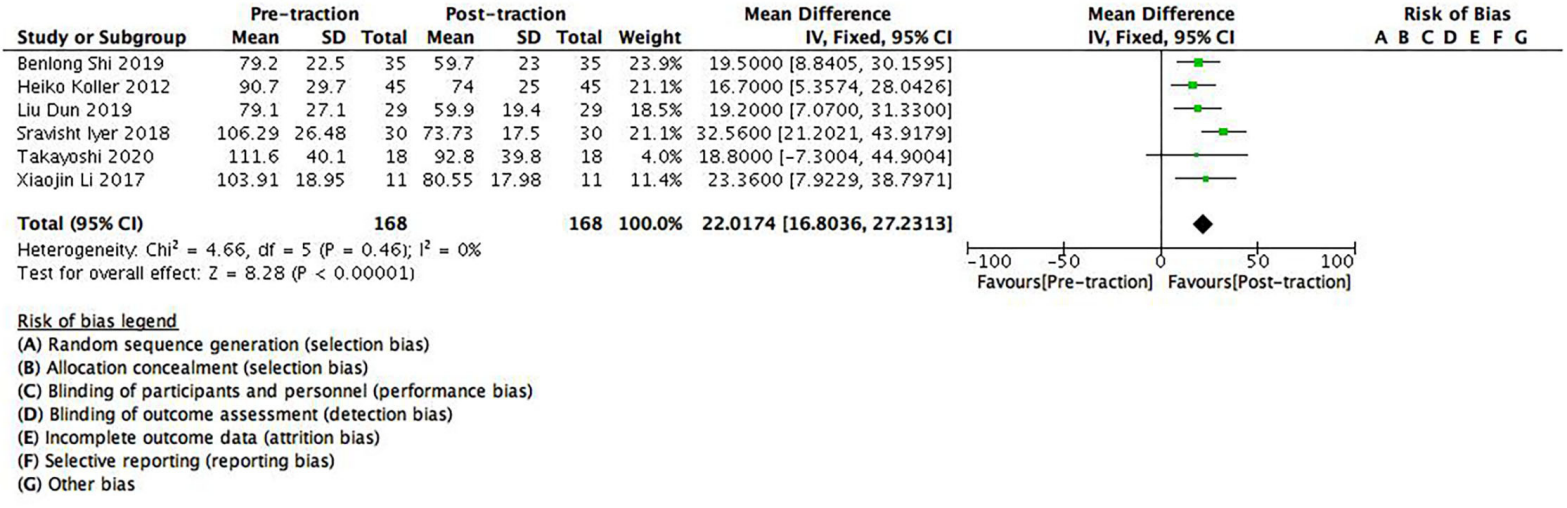
A forest plot depicting the changes in sagittal Cobb angle of scoliosis patients between pre- and post-traction measurements.

There were six studies containing 168 patients which provided data on pre- and post-traction sagittal Cobb angle. Significant differences were found between pre- and post-traction sagittal Cobb angle value [MD = 22.02 (95%CI 16.8–27.23), *p* < 0.001, *I*^2^ = 0%, Figure 3]. As no heterogeneity was found between the studies, a fixed-effects model was used for this parameter.

In addition, we also found significant differences between pre- and post-traction coronal balance [MD = 2.53 (95%CI 0.45–4.61), *p* < 0.05], sagittal balance [MD = 2.6 (95%CI 1.21–3.99), *p* < 0.01] and T1-S1 length value [MD = −59.10 (95%CI −109.13 to −9.07), *p* < 0.05) in this study.

### Pulmonary Function

**Figure 4:**
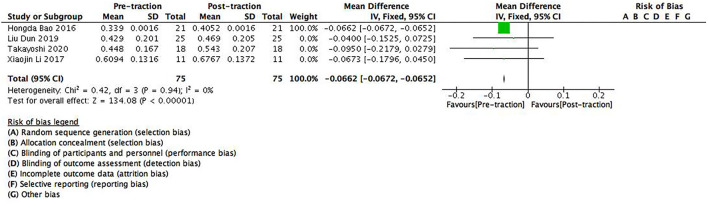
A forest plot depicting the changes in FVC predicted value (%FVC) of scoliosis patients between pre- and post-traction measurements.

The data of pre- and post-traction FVC predicted value (%FVC) was recorded in four studies containing 75 patients. Significant differences were found between pre- and post-traction %FVC [MD = −0.0662 (95%CI −0.0672–0.0652), *p* < 0.001, *I*^2^ = 0%, Figure 4]. A fixed-effects model was used because no heterogeneity was found between the studies for this parameter.

**Figure 5:**
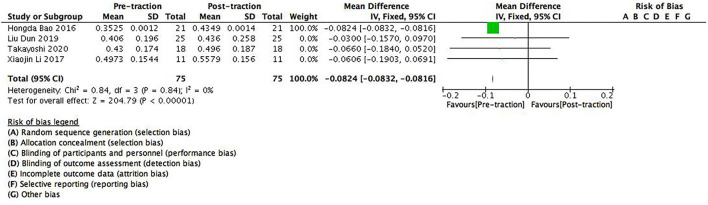
A forest plot depicting the changes in FEV1 predicted value (%FEV1) of scoliosis patients between pre- and post-traction measurements.

There were four studies containing 75 patients which provided data on pre- and post-traction FEV1 predicted value (%FEV1). Significant differences were found between pre- and post-traction %FEV1 [MD = −0.0824 (95%CI −0.0832–−0.0816), *p* < 0.001, *I*^2^ = 0%, Figure 5]. As no heterogeneity was found between these studies for this parameter, a fixed-effects model was used.

We also found significant differences between pre- and post-traction PEF predicted value [MD = −0.146 (95%CI −0.146–−0.144), *p* < 0.01] and FVC/FEV1 [MD = 0.0249 (95%CI 0.0244–0.0254), *p* < 0.01]. However, for FVC [MD = −0.0727 (95%CI −0.166–0.0210), *p* > 0.05), FEV1 [MD = −0.0801 (95%CI −0.174–0.0137), *p* > 0.05], FEV1/FVC [MD = −0.0171 (95%CI −0.0822–0.0480), *p* > 0.05] and PEF [MD = −0.59 (95%CI −1.26–0.0846), *p* > 0.05], there was no statistical significance between pre- and post-traction measurements on these indicators.

## Discussion

Severe spinal deformity with pulmonary insufficiency will lead to pulmonary heart disease, pulmonary hypertension, and other respiratory and circulatory complications. Those patients suffer from respiratory failure and heart failure, which seriously affect their quality of life. Previous studies have shown that one-stage surgery for severe spinal deformity with pulmonary insufficiency is not only risky but also difficult and ineffective in correcting scoliosis (14). The use of staged surgery can significantly improve the tolerance to the operation of patients, increase the correction rate of scoliosis, and reduce postoperative pulmonary complications. As a gradual traction method using the weight of the body as a counter-force, halo-gravity traction is safer than other traction methods, with a lower post-traction complication rate (15). Thus halo-gravity traction is often used preoperatively and staged surgery combined with preoperative halo-gravity traction not only straightens the curved spine of patients and simplifies operation, but also improves the cardiopulmonary functions of patients, helping the physician understand the tolerance of the spinal cord and the occurrence of spinal cord nerve injury in the traction state and reduces postoperative spinal cord, nerve, and other related complications. Currently, staged surgery combined with preoperative halo-gravity traction is one of the most effective methods to treat severe scoliosis patients with pulmonary insufficiency in clinical practice (9, 10).

Halo-gravity traction loosens the issue contractures around the spine, improves spine flexibility, and straightens the bent spine, thereby it can reduce the degree of scoliosis in a certain period of time. The results of this study also showed that halo-gravity traction could effectively reduce the degree of deformity [coronal Cobb angle: (95%CI 21.16–33.4), *p* < 0.001; sagittal Cobb angle: (95%CI 16.80–27.23), *p* < 0.001] in severe scoliosis patients with pulmonary insufficiency, especially decreases the Cobb angle. As for scoliosis patients ≥100° curves, our study showed that halo-gravity traction could reduce the degree of deformity of these patients to a certain extent (Figures 2, 3), which further supported the findings by the team of Kei Watanabe that the combination of halo-gravity traction and corrective fusion surgery could be used on scoliosis patients with ≥100° curves as safe and effective surgical treatments (16).

In this study, we found that halo-gravity traction could improve pulmonary function in patients with severe scoliosis having pulmonary insufficiency [%FVC: (95%CI −0.0672–−0.0652], *p* < 0.001; %FEV1: (95%CI−0.0832–−0.0816), p < 0.001]. The effect of halo-gravity traction on improving the pulmonary functions of patients was mainly due to its ability on stretching the spine, increase the volume of the thoracic cavity, and alleviate the compression of surrounding tissues. It should be noted that due to limited traction force, the effect of halo-gravity traction on increasing the thoracic volume was not as direct as straightening the spine, so the improvements on the Cobb angle of patients were more significant than on pulmonary functions in this study.

The improvements of some pulmonary function indicators (e.g., PEF, FVC, FEV1) were not statistically significant in this study as reported in other studies, it might be because halo-gravity traction had its disadvantages in the application, halo-gravity traction was not applied continuously for 24 h in some research cases, or the number of included studies was small.

The disadvantages of halo-gravity traction are small traction weight and unsuitability for long-term application. However, compared to other traction methods, halo-gravity traction is still the most commonly used preoperative traction method in clinical practice due to its convenience in operation, good effects in ameliorating the conditions of patients, the lower incidence of traction-related complications, and no extended periods of bed rest after traction (17).

Our systematic review and meta-analysis provided moderate-quality evidence that halo-gravity traction was an effective method to treat severe scoliosis patients with pulmonary insufficiency before surgical operation. It should also be noted that our meta-analysis exhibited various limitations. First, only seven studies were preserved for meta-analysis, the number of review papers and the number of patients included in this study was limited; factors that would affect the effect of halo-gravity traction such as age, the length of traction time were not fully discussed limited by the small number of studies included. Second, our study only analyzed pre- and post-traction data, long-term follow-ups were not included. Third, the incidence of postoperative complications and the improvement of the nutritional status of patients were needed to demonstrate the effects and advantages of preoperative halo-gravity traction more comprehensively and provide better references for follow-up clinical practice.

## Conclusions

The preoperative application of halo-gravity traction can effectively improve radiographic scoliosis and pulmonary function of severe scoliosis patients. The effectiveness of halo-gravity traction on the pulmonary function of patients was mainly reflected in the improvements of FVC and FEV1; however, the effectiveness on the whole pulmonary function of patients was only reflected in the change of numerical value. Therefore, preoperative application of halo-gravity traction can improve the degree of deformity and pulmonary function of severe scoliosis patients to some extent, improve the surgical tolerance of patients and reduce the occurrence of complications, which together increase the safety of surgery.

## Data Availability Statement

The original contributions presented in the study are included in the article/supplementary material, further inquiries can be directed to the corresponding authors.

## Author Contributions

YW and BJ: conception and design. ZY, YL, and JL: acquisition, analysis, and interpretation of data. ZY and YL: manuscript writing. LQ and SW: critical revision of the manuscript. All authors read and approved the final manuscript.

## Funding

This study is financially supported by the National Key R&D Program of China (No. 2020YFC1107601).

## Conflict of Interest

The authors declare that the research was conducted in the absence of any commercial or financial relationships that could be construed as a potential conflict of interest.

## Publisher's Note

All claims expressed in this article are solely those of the authors and do not necessarily represent those of their affiliated organizations, or those of the publisher, the editors and the reviewers. Any product that may be evaluated in this article, or claim that may be made by its manufacturer, is not guaranteed or endorsed by the publisher.

## Abbreviations

MD: Mean difference
CI: Confidence interval
FVC: Forced vital capacity
FEV1: Forced expiratory volume in one second
%FVC: Forced vital capacity predicted value
%FEV1: Forced expiratory volume in one second predicted value
MESH: Medical subject headings
AHRQ: Agency for healthcare research and quality
PEF: Peak expiratory flow.

## Appendix

**Table A1:** Quality assessment of the included studies.

**Reference**	**Quality assessment**			
	**Defining the source of information**	**Listing inclusion and exclusion criteria**	**Indicating time period used for identifying patients**	**Explaining any patient exclusions from analysis**
Shimizu et al. (13)	Yes	No	Yes	Yes
Shi et al. (12)	Yes	Yes	Yes	No
Iyer et al. (8)	Yes	No	Yes	Yes
Liu et al. (11)	Yes	Yes	Yes	Yes
Li et al. (10)	Yes	Yes	Yes	No
Koller et al. (9)	Yes	No	No	Yes
Bao et al. (7)	Yes	Yes	No	No

Appendix A Pubmed Search Strategy

(Scoliosis [Mesh]) AND ((traction [Title/Abstract]) OR (Halo-femoral traction [Title/Abstract]) OR (HFT[Title/Abstract]) OR (Halo-gravity traction [Title/Abstract]) OR (HGT[Title/Abstract]) OR (Halo-pelvic traction [Title/Abstract]) OR (HPT[Title/Abstract]))

Appendix B

Embase Search Strategy

([traction OR 'halo femoral'] AND (traction OR 'halo gravity') AND (traction OR hft OR hgt OR hpt OR 'halo pelvic') AND traction AND ('scoliosis'/exp OR scoliosis)]

Appendix C

The Cochrane Library Search Strategy

Scoliosis AND (Traction OR Halo-femoral traction OR HFT OR Halo-gravity traction OR HGT OR Halo-pelvic traction OR HPT)

Appendix D
